# Prior Clinico‐Radiological Features Informed Multi‐Modal MR Images Convolution Neural Network: A novel deep learning framework for prediction of lymphovascular invasion in breast cancer

**DOI:** 10.1002/cam4.6932

**Published:** 2024-01-17

**Authors:** Hong Zheng, Lian Jian, Li Li, Wen Liu, Wei Chen

**Affiliations:** ^1^ Department of Radiology, Hunan Cancer Hospital, The Affiliated Cancer Hospital of Xiangya School of Medicine Central South University Changsha Hunan China; ^2^ Department of Radiology Hunan Children's Hospital Changsha Hunan China; ^3^ Department of Radiology The Third Xiang Ya Hospital Central South University Changsha Hunan China; ^4^ Department of Radiology The Second People's Hospital of Hunan Province, Brain Hospital of Hunan Province Changsha Hunan China

**Keywords:** breast cancer, lymphovascular invasion, Prior Clinico‐Radiological Features Informed Channel Attention Neural Network, radiomics

## Abstract

**Background:**

Current methods utilizing preoperative magnetic resonance imaging (MRI)‐based radiomics for assessing lymphovascular invasion (LVI) in patients with early‐stage breast cancer lack precision, limiting the options for surgical planning.

**Purpose:**

This study aimed to develop a sophisticated deep learning framework called “Prior Clinico‐Radiological Features Informed Multi‐Modal MR Images Convolutional Neural Network (PCMM‐Net)” to improve the accuracy of LVI prediction in breast cancer. By incorporating multiparameter MRI and prior clinical knowledge, PCMM‐Net should enhance the precision of LVI assessment.

**Methods:**

A total of 341 patients with breast cancer were randomly divided into training and validation groups at a ratio of 7:3. Imaging features were extracted from T1‐weighted, T2‐weighted, and contrast‐enhanced T1‐weighted MRI sequences. Stepwise univariate and multivariate logistic regression were employed to establish a clinico‐radiological model for LVI prediction. The radiomics model was built using redundancy and the least absolute shrinkage and selection operator. Then, two deep learning frameworks were developed: the Multi‐Modal MR Images Convolutional Neural Network (MM‐Net), which does not consider prior radiological features, and PCMM‐Net, which incorporates multiparameter MRI and prior clinical knowledge. Receiver operating characteristic curves were used, and the corresponding areas under the curves (AUCs) were calculated for evaluation.

**Results:**

PCMM‐Net achieved the highest AUC of 0.843. The clinico‐radiological features displayed the lowest AUC value of 0.743, followed by MM‐Net with an AUC of 0.774, and radiomics with an AUC of 0.795.

**Conclusions:**

This study introduces PCMM‐Net, an innovative deep learning framework that integrates prior clinico‐radiological features for accurate LVI prediction in breast cancer. PCMM‐Net demonstrates excellent diagnostic performance and facilitates the application of precision medicine.

## 1 INTRODUCTION

Breast cancer is the most common malignancy with historically high incidence rates among women in Europe and the United States; its incidence has steadily increased in recent years, particularly in young individuals.^1^ At the time of diagnosis, approximately 5% of breast cancers have already metastasized, and 20%–30% of localized breast cancers develop distant metastases.^2^ Metastases remain the primary cause of breast cancer‐related mortality and pose significant challenges to measures aimed to reduce the mortality rate. Despite advancements in breast cancer treatment, patients with distant metastases or invasion of adjacent organs have low survival rates.^3^ Recent clinicopathological studies have identified a correlation between lymphovascular invasion (LVI) and regional and systemic lymph node metastases, suggesting that LVI plays a crucial role in the treatment of breast cancer.^4^ In addition, LVI often precludes breast‐conserving surgery.^5^ Therefore, accurately determining LVI status before surgery, although challenging, is crucial.

Previous studies have indicated that tumor features on quantitative radiomics can be used to predict LVI. Specifically, in patients with breast cancer, magnetic resonance imaging (MRI)‐based radiomics is a promising method for the accurate prediction of LVI status^6^, ^7^, ^8^, ^9^; however, it has limitations. The reproducibility and accuracy of lesion segmentation are susceptible to manual errors and may be influenced by the expertise of the radiologists. Furthermore, radiomics analysis is often laborious and time‐consuming and involves tasks such as segmentation, feature extraction, and subsequent selection.^10^, ^11^ Therefore, the development of a more objective, precise, and convenient approach for the accurate assessment of the LVI status is imperative.

Recent advancements in artificial intelligence offer potential solutions to resolve certain limitations of medical imaging. Deep learning, a machine learning technique, has shown promise as a method to aid diagnosis, discover new features, and predict patient outcomes.^12^, ^13^ However, previous studies have primarily focused on image segmentation masks while neglecting the incorporation of MR radiological features relevant to the LVI status. Research has reported that MR radiological features such as the rim sign seen on diffusion‐weighted imaging (DWI), subcutaneous edema, peritumoral edema, the adjacent vessel sign (AVS), and MR‐reported axillary lymph node (mrALN) involvement are associated with LVI.^14^, ^15^, ^16^, ^17^, ^18^

To date, some studies have integrated clinico‐radiological features with deep learning or radiomic features. Zhao et al. proposed a cross‐modal deep learning system which can successfully incorporate prior clinical knowledge and CT images into a 3D neural network to predict lymph node metastasis.^19^ Zheng et al.^20^ suggested clinical parameter combined deep learning radiomics (DLR) of conventional ultrasound and shear wave elastography of breast cancer for preoperatively predicting axillary lymph node status in patients with early‐stage breast cancer. Support vector machine models based on radiomic and deep features extracted from multiparametric MRI were also reported.^21^ These methods fused prior clinico‐radiological features with image features into vector inputs for classifier learning, but have not been used for LVI prediction. Therefore, further exploration to enhance the understanding and utilization of these features in medical imaging research is necessary.

In the present study, we propose a novel predictive framework called the Prior Clinico‐Radiological Features Informed Multi‐Modal MR Images Convolution Neural Network (PCMM‐Net). This framework integrates T1‐weighted (T1WI), T2‐weighted (T2WI), and dynamic contrast‐enhanced (DCE)‐MRI and 16 images filled by 16 prior clinico‐radiological features to accurately evaluate the risk of LVI in breast cancer patients. The most significant difference between our method and existing methods of fusing prior clinico‐radiological features is that our model uses prior clinico‐radiological features as image inputs from the beginning instead of fusing them with image features into vector inputs for classifier learning. It was significant to mention that learning prior clinico‐radiological features as image inputs were first employed for LVI prediction. Additionally, the incorporation of 3 modal images and 16 images filled by 16 prior clinical features is a novel approach of utilizing prior radiological information. We also compared the diagnostic performance of clinico‐radiological features, multiparameter MR‐based radiomics, the Multi‐Modal MR Images Convolution Neural Network (MM‐Net), which does not incorporate prior radiological features, and PCMM‐Net. The primary objective of this study was to address the existing gap in reliable assessment methods and establish a preliminary research foundation for the development of artificial intelligence algorithms aimed at predicting LVI in breast cancer.

## 2 MATERIALS AND METHODS

### 2.1 Study population

This retrospective study was approved by the Institutional Ethics Review Board of our hospital. The requirement for informed consent was waived because of the retrospective nature of the study. We included 341 consecutive female patients with confirmed invasive breast cancer who underwent pretreatment contrast‐enhanced MRI at our hospital between January 2019 and June 2023.

The inclusion criteria were as follows: (1) visible primary breast lesions on MRI, (2) newly diagnosed invasive breast carcinoma confirmed through histopathological evaluation of surgical specimens, and (3) mastectomy or lumpectomy within 14 days of the MRI examination. Patients who underwent breast lesion biopsy before MRI examination or received neoadjuvant chemotherapy before surgery were excluded. Patients with significant artifacts on MR images were also excluded to ensure accurate and reliable results.

**FIGURE 1:**
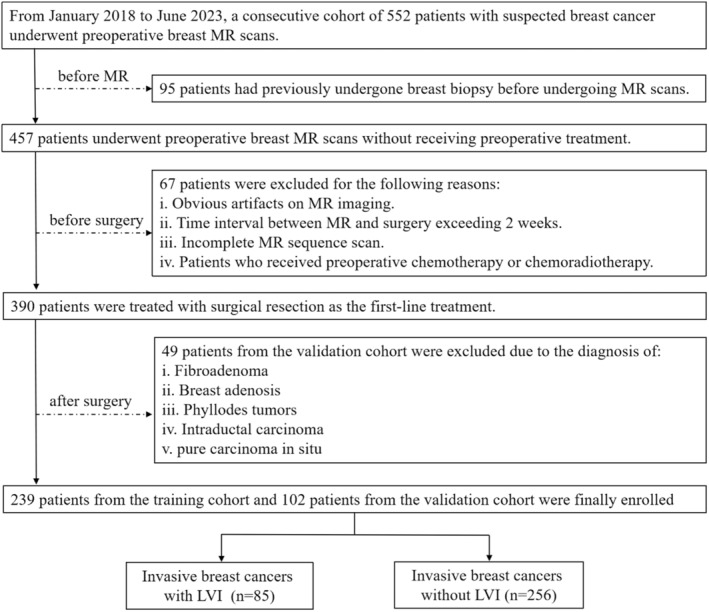
Flowchart of the study enrollment process.

Training and validation datasets were created using random stratified sampling. There were 239 patients (180 LVI‐negative and 59 LVI‐positive) in the training dataset and 102 patients (76 LVI‐negative and 26 LVI‐positive) in the validation dataset (ratio, 7:3). Figure 1 shows the patient selection process.

### 2.2 MRI examination

To minimize variations in image quality across cases, we included only breast MRI studies acquired on a 1.5 Tesla MAGNETOM Aera scanner (Siemens Healthineers, Erlangen, Germany). All MRI examinations were conducted using a 1.5 Tesla MAGNETOM Aera scanner (Siemens Healthineers, Erlangen, Germany) equipped with an 8‐channel phased‐array breast surface coil. The imaging protocol consisted of three main sequences: turbo spin‐echo T1‐weighted imaging, fat‐suppressed spin‐echo T2‐weighted imaging, dynamic contrast‐enhanced MRI, and DWI. Specifically, we selected early‐phase contrast‐enhanced T1‐weighted images (cT1WI) and fat‐suppressed T1‐weighted fast low‐angle shot 3D DCE images. The imaging parameters for this sequence were as follows: repetition time = 4.3 ms, echo time = 1.4 ms, 12°, field of view = 200 × 320 mm, matrix size = 307 × 512, and slice thickness = 1.5 mm. During the examination, a gadolinium‐based contrast agent was administered at a dose of 0.2 mmol/kg using a mechanical power injector, followed by a saline flush of 15–20 mL. The imaging parameters for DWI were as follows: repetition time = 8500 ms, echo time = 70 ms, field of view = 250 × 330 mm, matrix size = 192 × 192, number of excitations = 1, slice thickness = 3 mm, intersection gap = 1 mm, and *b*‐values = 0 and 1000 s/mm^2^.

### 2.3 Radiologic evaluation and modeling

The MR images were evaluated by experienced radiologists following the American College of Radiology Breast Imaging Reporting and Data System criteria.^22^ These criteria standardize the classification of breast imaging findings, ensuring the consistent assessment and reporting of radiological features. Two radiologists conducted blinded clinical and laboratory data reviews to ensure unbiased evaluation. In cases of disagreement, an agreement was reached through negotiation.

Radiologists assessed various radiological features, including fibroglandular tissue density, time‐signal intensity curves, breast parenchymal enhancement, peritumoral edema, subcutaneous edema, intratumoral high signal intensity, DWI rim sign, AVS, and internal enhancement patterns.

Peritumoral edema refers to an increased fat‐suppressed T2‐weighted signal intensity appearing as bright as water around the tumor mass.^15^, ^16^ The radiologists also examined the images for subcutaneous edema, skin thickening, and high signal intensity in the subcutaneous tissue.^15^ High intratumoral signal intensity was defined as a region of high signal intensity within the tumor relative to the surrounding breast tissue, visualized on fat‐suppressed T2‐weighted images.^16^ The DWI rim sign is a high peripheral signal outlining >90% (complete) or < 90% (incomplete) of the lesion on DWI.^14^ Other characteristics, such as the AVS and increased ipsilateral vascularity, were also considered.^18^ Moreover, the radiologists classified the internal enhancement patterns of lesions as homogeneous, heterogeneous, or rim enhancement.^18^ mrALNs were identified when the short‐axis diameter was larger than 10 mm, when the ratio of the longest axis to the shortest axis was <1.5, when there was loss of fatty hilum, or when there was eccentric cortical thickening.^17^

Univariate logistic regression analysis was conducted on the clinico‐radiological features. In addition, multivariate logistic regression analysis was performed on the clinico‐radiological features with a *p* < 0.1 in the univariate analysis.

### 2.4 MRI segmentation and radiomics feature extraction and reduction

A board‐certified breast radiologist with extensive experience manually delineated the 3D volume of interest (VOI) around the tumor. This process involved outlining the tumor on transverse cT1WI images using a 3D Slicer (version 4.11.0; https://www.slicer.org). Subsequently, the 3D Slicer was used to register the T1WI and T2WI images into cT1WI images and share the VOIs.

For image preprocessing and feature extraction, we used Pyradiomics (version 3.0) implemented in Python. We calculated the first order and intensity histogram statistics, generated 2D and 3D shape descriptors, and computed texture features (e.g., gray‐level dependence and size zone matrices). Prior to feature selection, patient feature values underwent *z*‐score normalization.

**FIGURE 2:**
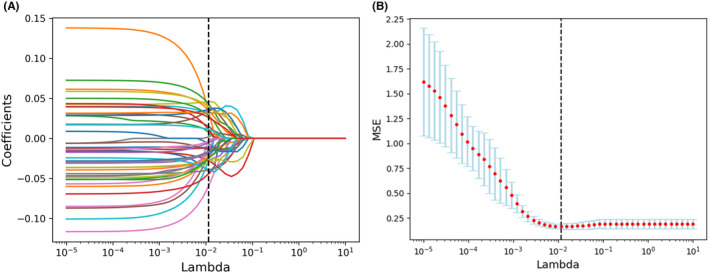
Feature selection for the least absolute shrinkage and selection operator (LASSO) logistic regression and the predictive accuracy of the radiomics signature. (A) LASSO coefficient profiles for all features. (B) Selection of tuning parameter (λ) using fivefold cross‐validation with minimum criteria.

To ensure the selection of robust radiomics features, a three‐step procedure was implemented. First, a univariate analysis was conducted to identify features related to LVI, with a significance threshold of *p* < 0.01. Subsequently, Spearman's or Pearson's correlation analyses were performed to eliminate redundant features that exhibited a correlation coefficient (r) of ≥0.90.^7^ Finally, we employed the least absolute shrinkage and selection operator (LASSO) method for feature selection and regularization (Figure 2). This method aims to improve the accuracy and interpretability of a model by effectively identifying relevant features.

Note that the radiomics model after dimensionality reduction using LASSO was not our primary focus, as it was simply a method for comparison. Instead, we focused on the deep learning framework integrating prior clinico‐radiological features for the precise prediction of LVI. While Pyradiomics uses mathematical formulas to extract (semi‐)quantitative features from medical images, the features extracted by the 3D neural network are automatically learned by the network, which provides a more abstract and higher‐level representation of the original image.

### 2.5 Development of the deep learning model

An appropriate cross‐modal merging technique was required to combine the different sources of information (i.e., images and clinical texts). We propose an end‐to‐end deep learning architecture called PCMM‐Net, which can fuse prior clinico‐radiological features and multi‐modal images. We employed a 3D residual channel attention‐based backbone to extract the image features.^23^, ^24^ The inputs to the proposed model were three cubic patches from three‐phase postcontrast DCE‐MRI scans and 16 images filled by 16 prior clinico‐radiological features. Values less than zero in the DCE‐MRI scans were set to zero, values above zero were scaled 0–1. Three cubic patches from the volumes under the VOIs measuring 32 × 32 × 32 mm were cropped with the same center as the VOI from the three‐phase post‐contrast DCE‐MRI.

**FIGURE 3:**
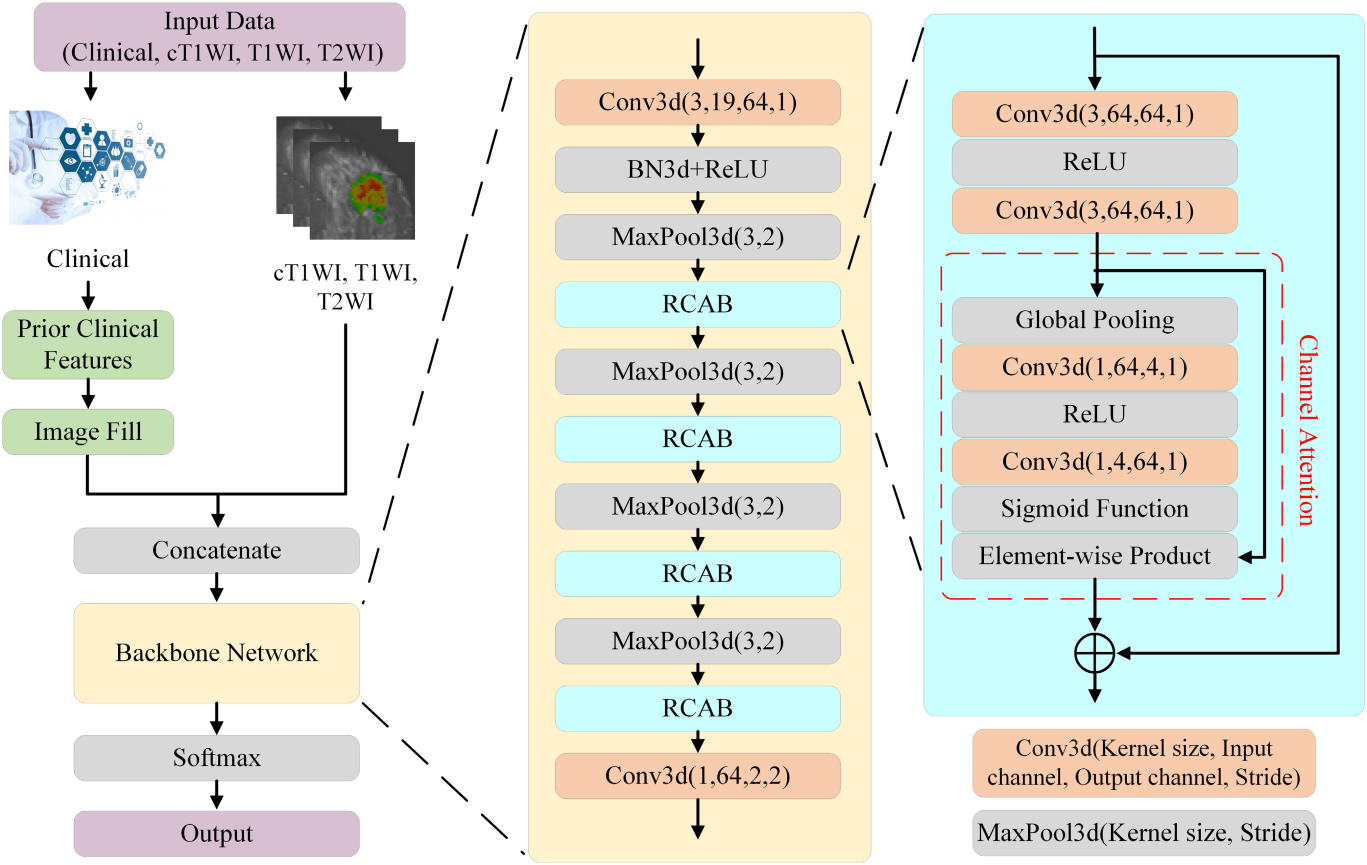
Architecture of the proposed Prior Clinico‐Radiological Features Informed Multi‐Modal MR Images Convolutional Neural Network (PCMM‐Net).

The proposed PCMM‐Net includes two stages. First, 16 images filled by the prior clinico‐radiological features were merged with the three cubic patches from three‐phase postcontrast DCE‐MRI scans. The second stage automatically extracted the image features associated with the LVI status using the backbone network. Then, a prediction was made (i.e., LVI‐positive or LVI‐negative). The architecture of the proposed PCMM‐Net is shown in Figure 3 and further details are provided below.

In the second stage, because the available medical data were limited, the backbone network had to be compact and have few parameters to simplify the training procedure. ResNet and channel attention satisfy these requirements and have been used to achieve breakthrough results in image classification.^23^, ^24^, ^25^, ^26^ The architecture of the backbone network mainly consisted of four 3D residual channel attention blocks (RCAB)^25^ comprising convolution, rectified linear units (ReLU), channel attention, and skip connections, which were employed to produce hierarchical features related to LVI status. The attention mechanism automatically determined the connections between the feature maps and the hierarchy of significance for the final goal. The backbone network contained a head block and a tail block. The head block included a convolution layer, batch normalization layer (BN3d), ReLU, and max pooling layer (MaxPool3d). The input volumes were transformed into feature maps by increasing the number of channels from 19 to 64 and decreasing the volume scale of the convolutional layer. BN3d^27^ was used to reduce the internal covariance shift, and the ReLU reduced the probability of the vanishing gradient. MaxPool3d was used to compress the data and parameters to reduce overfitting. The tail block is a convolution layer to reduce the dimensions of the hierarchical features extracted by fusing the spatial features. Finally, softmax activation was used to determine the LVI risk probability.

The training data for the proposed PCMM‐Net were augmented by random flipping along three volume directions. We directly trained our PCMM‐Net from scratch. A widely used cross‐entropy loss function was employed to train the network. Because of the imbalance between LVI‐negative and LVI‐positive data, we set the loss coefficients of LVI‐negative and LVI‐positive to 0.25 and 0.75, respectively. PyTorch 1.6.0 and Python 3.7.7 were used to implement the proposed PCMM‐Net. We employed the Adam^28^ optimization with a learning rate of 0.0001 and a batch size of 48 to train the network for 100 epochs and ensure network convergence. The final model was selected based on the least amount of loss seen in the training dataset. All the deep learning methods were executed on a workstation with a Xeon CPU E5‐2630 (Intel, Santa Clara, CA, USA) and GeForce GTX 1080 Ti (NVIDIA, Santa Clara, CA, USA).

### 2.6 Statistical analyses and model evaluation

In this study, training datasets were used for both model training and parameter tuning. Subsequently, validation datasets were employed to evaluate the final model performance. All data processing and statistical analyses were performed using Python version 3.7.7. The scikit‐learn 0.23.2 package was used, specifically the confusion matrix function to generate confusion matrices for evaluating the performance of prior clinico‐radiological features, multiparameter MR‐based radiomics, MM‐Net, and PCMM‐Net. Receiver operating characteristic (ROC) curves were used to calculate the areas under the curves (AUCs) for all models. The AUC, sensitivity, and specificity were used to assess the performance of the models. The significance of the difference between the PCMM‐Net model and other models was evaluated using the DeLong method.

## 3 RESULTS

### 3.1 Examined clinico‐radiological features

Within the statistical power of this study, no statistically significant differences were observed in the clinico‐radiological features between the training and validation datasets (Table S1). Table 1 presents a comprehensive analysis of the clinico‐radiological features for patients categorized into the LVI‐positive and LVI‐negative groups in the training dataset.

**TABLE 1:** Comparative analysis of clinico‐radiological features between LVI‐negative and ‐positive cases.

Variables	Total (*n* = 239)	LVI‐negative (*n* = 180)	LVI‐positive (*n* = 59)	*p*‐value
Age, Median (Q1, Q3)	52 (45, 58.5)	52 (45, 59)	51 (44, 57)	0.491
Menopausal status, *n* (%)				0.976
Premenopausal	111 (46.4)	83 (46.1)	28 (47.5)	
Postmenopausal	128 (53.6)	97 (53.9)	31 (52.5)	
Location, *n*(%)				0.166
Left	126 (52.7)	100 (55.6)	26 (44.1)	
Right	113 (47.3)	80 (44.4)	33 (55.9)	
TIC curves, *n* (%)				0.386
Type 1	11 (4.6)	8 (4.4)	3 (5.1)	
Type 2	94 (39.3)	75 (41.7)	19 (32.2)	
Type 3	134 (56.1)	97 (53.9)	37 (62.7)	
FGT density, *n* (%)				0.156
Dense	50 (20.9)	42 (23.3)	8 (13.6)	
Heterogeneously dense	86 (36)	59 (32.8)	27 (45.8)	
Scattered	73 (30.5)	54 (30)	19 (32.2)	
Predominantly fatty	30 (12.6)	25 (13.9)	5 (8.5)	
BPE, *n* (%)				0.960
None/minimal	64 (26.8)	49 (27.2)	15 (25.4)	
Mild	104 (43.5)	79 (43.9)	25 (42.4)	
Moderate	47 (19.7)	34 (18.9)	13 (22)	
Marked	24 (10)	18 (10)	6 (10.2)	
Intratumoral high signal intensity, *n* (%)				0.803
Absence	165 (69)	123 (68.3)	42 (71.2)	
Presence	74 (31)	57 (31.7)	17 (28.8)	
Peritumoral edema, *n* (%)				<0.001
Absence	163 (68.2)	143 (79.4)	20 (33.9)	
Presence	76 (31.8)	37 (20.6)	39 (66.1)	
Subcutaneous edema, *n* (%)				0.401
Absence	197 (82.4)	151 (83.9)	46 (78)	
Presence	42 (17.6)	29 (16.1)	13 (22)	
Intratumoral necrosis, *n* (%)				0.593
Absence	194 (81.2)	148 (82.2)	46 (78)	
Presence	45 (18.8)	32 (17.8)	13 (22)	
Internal enhancement pattern, *n* (%)				0.156
Homogeneous	192 (80.3)	149 (82.8)	43 (72.9)	
Heterogeneous	45 (18.8)	30 (16.7)	15 (25.4)	
Rim enhancement	2 (0.8)	1 (0.6)	1 (1.7)	
Adjacent vessel sign, *n* (%)				0.033
Absence	95 (39.7)	79 (43.9)	16 (27.1)	
Presence	144 (60.3)	101 (56.1)	43 (72.9)	
Increased ipsilateral vascularity, *n* (%)				0.683
Absence	125 (52.3)	96 (53.3)	29 (49.2)	
Presence	114 (47.7)	84 (46.7)	30 (50.8)	
mrALN status, *n* (%)				0.670
Absence	189 (79.1)	144 (80)	45 (76.3)	
Presence	50 (20.9)	36 (20)	14 (23.7)	
Short‐axis diameter of largest ALN, Median (Q1, Q3)	0.5 (0.3, 0.8)	0.5 (0, 0.8)	0.5 (0.4, 0.8)	0.257
DWI rim sign, *n* (%)				<0.001
Absence	177 (74.1)	147 (81.7)	30 (50.8)	
Presence	62 (25.9)	33 (18.3)	29 (49.2)	

Abbreviations: BPE, breast parenchymal enhancement; DWI, diffusion weighted imaging; FGT, fibroglandular tissue; LVI, lymphovascular invasion; mrALN, MRI‐reported axillary lymph node; TIC, time‐signal intensity.

**FIGURE 4:**
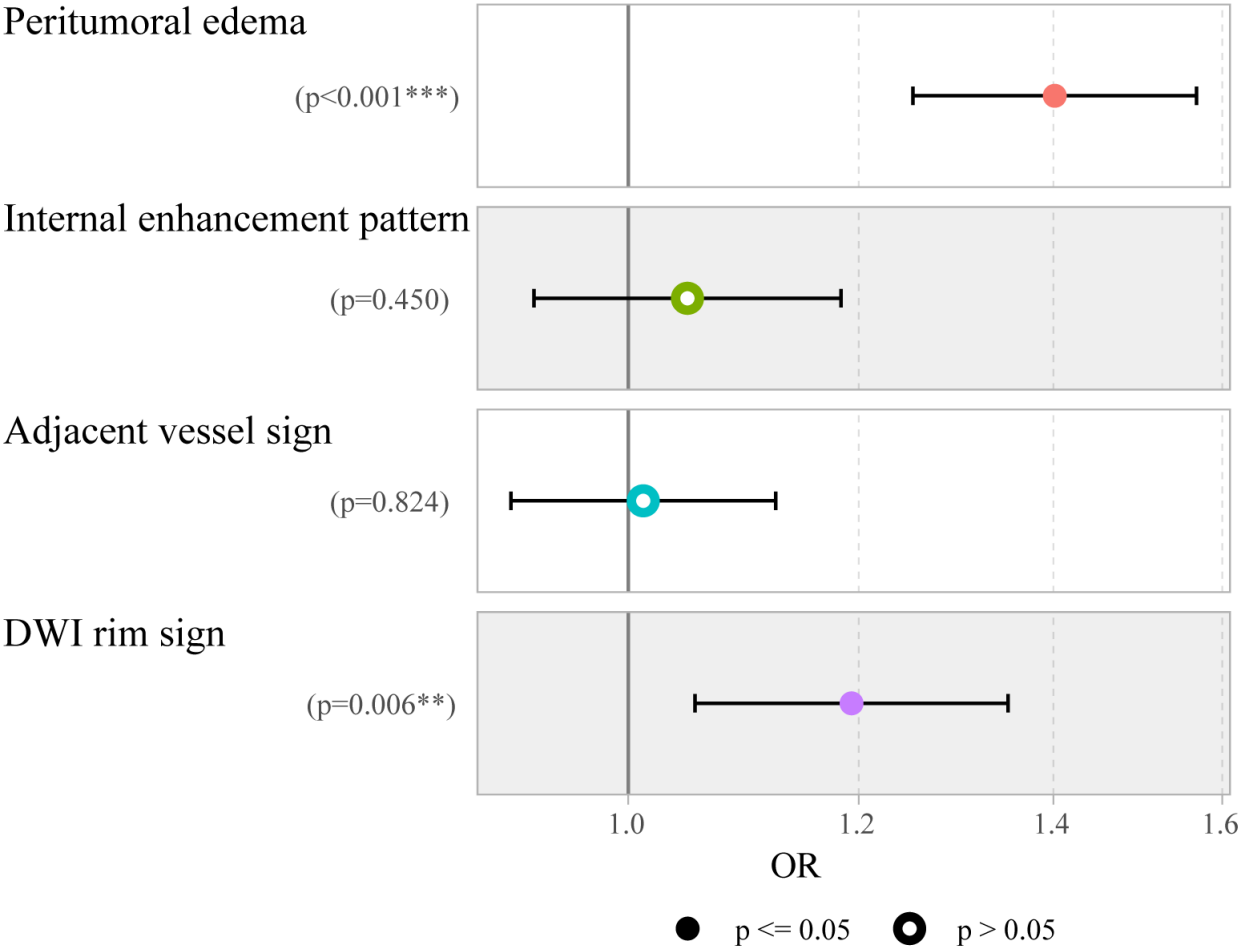
Forecast plot showing clinico‐radiological features for predicting lymphovascular invasion (LVI).

During the univariate logistic regression analysis, five variables were identified to be related to LVI positivity. Ultimately, peritumoral edema [odds ratio (OR), 1.401(1.253–1.568); *p* < 0.001] and the rim sign on DWI (OR, 1.193(1.054–1.351); *p* = 0.006) were included in the multivariate logistic regression analysis, as shown in Table 2 and Figure 4.

**TABLE 2:** Univariate and multivariate logistic analysis of clinico‐radiological features for evaluating LVI in breast cancer cases.

Variables	Univariate		Multivariate	
	Odd Ratio (95% CI)	*p*‐Value	Odd Ratio (95% CI)	*p*‐Value
Age	1 (0.97–1)	0.84		
Menopausal status	0.95 (0.53–1.7)	0.86		
Location	1.6 (0.88–2.9)	0.13		
TIC curves	1.3 (0.76–2.2)	0.35		
FGT density	1 (0.74–1.4)	0.94		
BPE	1.1 (0.77–1.5)	0.7		
Intratumoral high signal intensity	0.87 (0.46–1.7)	0.68		
Peritumoral edema	7.5 (3.9–14)	<0.001	1.401(1.253–1.568)	<0.001
Subcutaneous edema	1.5 (0.71–3.1)	0.3		
Intratumoral necrosis	1.3 (0.63–2.7)	0.47		
Internal enhancement pattern	1.8 (0.92–3.3)	0.087	1.048(0.928–1.183)	0.450
Adjacent vessel sign	2.1 (1.1–4)	0.024	1.012(0.911–1.124)	0.824
Increased ipsilateral vascularity	1.2 (0.66–2.1)	0.58		
mrALN status	1.2 (0.62–2.5)	0.54		
Short‐axis diameter of largest ALN	1.1 (0.93–1.2)	0.41		
DWI rim sign	4.3 (2.3–8.1)	<0.001	1.193(1.054–1.351)	0.006

Abbreviations: BPE, breast parenchymal enhancement; DWI, diffusion weighted imaging; FGT, fibroglandular tissue; LVI, lymphovascular invasion; mrALN, MRI‐reported axillary lymph node; TIC, time‐signal intensity.

### 3.2 Radiomics model construction

**FIGURE 5:**
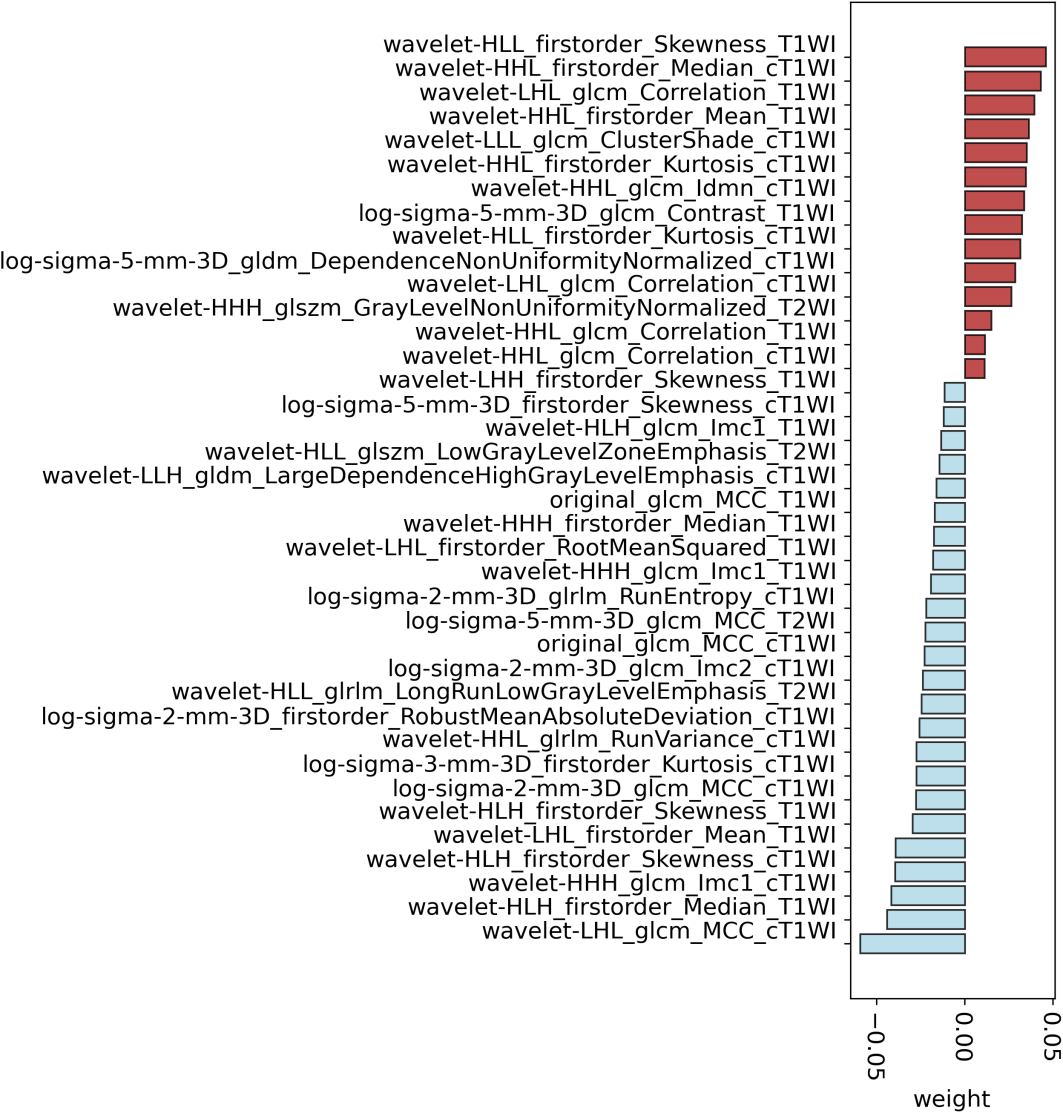
Distribution of the selected radiomics features by least absolute shrinkage and selection operator (LASSO).

In total, 3717 quantitative features were extracted from the transverse T1WI, T2WI, and cT1WI images (1239 features respectively). Redundancy was eliminated through univariate and Spearman's or Pearson's correlation analyses, resulting in 319 remaining features. These features were further evaluated using the LASSO method, resulting in a final set of 38 T1WI, seven cT1WI, and three T2WI features (Figure 5). The predicted probabilities of the radiomics model could be utilized to assess the risk of LVI and contribute to further evaluation.

### 3.3 Comparison of diagnostic performance among different models

**FIGURE 6:**
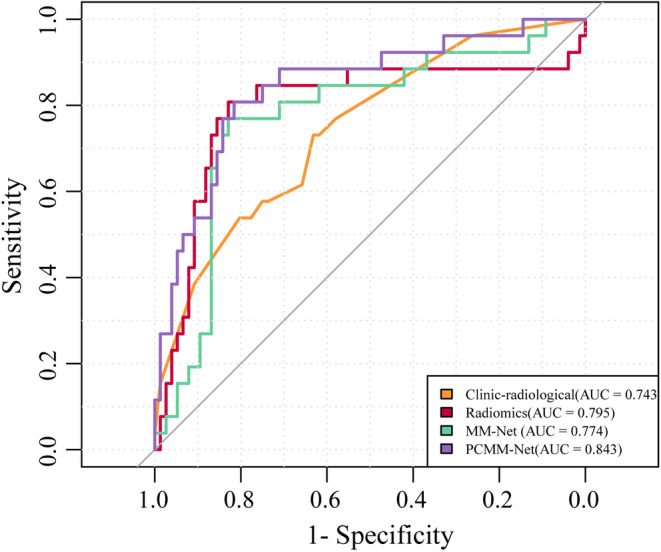
Receiver operating characteristic (ROC) curves for prediction performance of clinico‐radiological features, radiomics, Multi‐Modal MR Images Convolutional Neural Network (MM‐Net), and Prior Clinico‐Radiological Features Informed MM‐Net (PCMM‐Net).

Table 3 presents the discriminative abilities of the models, and Figure 6 exemplifies the ROC comparisons. PCMM‐Net exhibited excellent performance, surpassing that of the clinico‐radiological features (significant via Delong test, *p* = 0.028), MM‐Net (significant via Delong test, *p* = 0.043), and radiomics (significant via Delong test, *p* = 0.021). Notably, PCMM‐Net achieved a remarkable AUC of 0.843, accompanied by an accuracy(ACC) of 0.824, sensitivity of 0.818, and specificity of 0.816.

**TABLE 3:** Comparisons of Diagnostic Efficiency across Radiomics, MM‐Net, and PCMM‐Net modes.

Model	AUC	ACC	Sensitivity	Specificity	*p*‐Value
Clinic‐radiological	0.743	0.657	0.731	0.632	0.028
Radiomics	0.795	0.820	0.808	0.829	0.021
MM‐Net	0.774	0.814	0.769	0.829	0.043
PCMM‐Net	0.843	0.824	0.818	0.816	‐

*Note*: The *p*‐value signifies the contrast of AUCs between PCMM‐Net and the clinico‐radiological model, as well as between PCMM‐Net and Clinic‐radiological, and further between PCMM‐Net and MM‐Net.

Abbreviations: AUC, area under the curve; ACC, Accuracy; MM‐Net, Multi‐Modal MR Images Convolution Neural Network; PCMM‐Net, Prior Clinico‐Radiological Features Informed Multi‐Modal MR Images Convolution Neural Network.

In contrast, the clinico‐radiological features had the lowest AUC value of 0.743, alongside an ACC of 0.657, sensitivity of 0.731, and specificity of 0.632. Similarly, MM‐Net had an AUC of 0.774, ACC of 0.814, sensitivity of 0.769, and specificity of 0.829. The radiomics model exhibited slightly better values with an AUC of 0.795, ACC of 0.820, sensitivity of 0.808, and specificity of 0.829.

## 4 DISCUSSION

The present study introduces PCMM‐Net, a novel deep learning framework that integrates prior radiological features for LVI prediction in breast cancer risk assessment. PCMM‐Net demonstrated a slightly higher diagnostic performance and higher sensitivity than the radiomics model. In addition, the end‐to‐end design of PCMM‐Net outperformed the clinico‐radiological features assessed by radiologists.

Previous studies have investigated the various clinico‐pathological features related to LVI in breast cancer. These features include the rim sign on DWI, subcutaneous edema, peritumoral edema, AVS, and mrALN involvement.^14^, ^15^, ^16^, ^17^, ^18^ In contrast, our study focused on conducting multivariate logistic regression analysis to identify the most independent predictors. We found that peritumoral edema and the rim sign on DWI exhibited adequate predictive power. Peritumoral edema is believed to be caused by increased vascular permeability and the release of cytokines into the surrounding tissues.^15^, ^29^ Interestingly, Uematsu et al. proposed that edema may result from lymphatic drainage obstruction and dilation due to tumor emboli, and they observed a significant correlation between a high degree of LVI and the presence of edema.^15^ Zhang et al. developed a radiomics nomogram model incorporating peritumoral edema, which demonstrated promising and satisfactory calibration and discrimination abilities to predict LVI.^7^ Furthermore, the presence of the rim sign on DWI of breast lesions has been associated with malignancy and correlates with histological characteristics, such as the tumor grade, size, and subtype. The rim sign is also correlated with Ki‐67, a protein that indicates the cell proliferation rate and is a marker of cancer aggressiveness.^14^, ^18^

Radiomics models, which extract quantitative radiomic features from MR images, offer a clear advantage over clinico‐radiological features for the evaluation of tumor imaging phenotypes. These models can identify additional heterogeneous factors that may not be visually discernible. In the present study, the radiomics model demonstrated a significant predictive performance by achieving a higher AUC (0.795) than the clinico‐radiological features (0.743).

Moreover, we used deep learning techniques to predict the LVI status. While MM‐Net achieved a lower AUC of 0.774 than the radiomics model, our proposed PCMM‐Net (AUC of 0.843) surpassed all other models, showcasing its superiority in LVI prediction. The exceptional performance of PCMM‐Net highlights the importance of incorporating both clinical and radiological features to enhance the predictive capability for LVI prediction. This finding substantiates the significance of integrating multimodal information to improve the accuracy and reliability of prediction models in clinical practice. Additionally, the significant differences in performance metrics between PCMM‐Net and the other models further reinforce the potential applicability of our proposed approach in assisting medical professionals in making more informed decisions. It is noteworthy that PCMM‐Net demonstrated a sensitivity of 0.818 and specificity of 0.816, indicating its comprehensive capabilities. This can be attributed to PCMM‐Net's ability to automatically extract intrinsic features associated with LVI, eliminating the need for manually designed features used in traditional radiomics. Furthermore, PCMM‐Net integrates prior clinico‐radiological features, enabling comprehensive interpretation of tumor imaging phenotypes at a deeper and multi‐dimensional level. Consequently, the neural network can automatically select and adjust the weights of clinical and imaging features, equivalent to a feature selection procedure before model building. Moreover, PCMM‐Net is an end‐to‐end model where all features are trained together as inputs, which is more convenient than the multi‐step process of feature selection, ranking, and training.

This study had several limitations which should be alleviated in future work. Firstly, the retrospective design and the limited sample size restrict the generalizability of the findings. To address this, our aim is to expand the dataset used in our study, thereby increasing its generalizability and robustness. This expansion would include a larger and more diverse cohort of patients, allowing us to validate our findings across different populations and clinical settings. In addition, conducting a longitudinal study would provide valuable insights into the stability and performance of PCMM‐Net over time, enhancing its applicability for long‐term prognoses and treatment response monitoring. Secondly, the inclusion of data from only a single center may have introduced selection bias. To mitigate this, we plan to utilize external validation methods that can reduce data selection bias and enhance the generalizability of our results. Thirdly, existing publicly available benchmark datasets do not align with the same LVI labels and the three modal images (cT1WI, T1WI, and T2WI) used in our study. Furthermore, the detailed clinical information associated with radiological features was not fully documented. To address these concerns, it is crucial to explore the interpretability and explicatory ability of PCMM‐Net. Gaining an understanding of the underlying features and mechanisms that the model relies on will foster trust and facilitate its integration into routine clinical practice. In future studies, we intend to fully consider the use of existing public benchmark datasets to enhance the generalizability of our findings. Additionally, integrating other data modalities, such as genomics or histopathology, has the potential to elucidate the biological basis of the identified imaging features and improve the overall accuracy of the model. Continued research in these areas promises invaluable insights and advancements toward the development of more advanced and clinically meaningful predictive models.

## 5 CONCLUSIONS

By surpassing all other models, PCMM‐Net represents a significant breakthrough in terms of LVI prediction accuracy, resulting in an impressive AUC of 0.843. This advanced model holds immense potential for enhancing clinical decision‐making. The impressive results obtained in our study reinforce the effectiveness and reliability of PCMM‐Net as a tool for LVI prediction. Furthermore, our comprehensive performance analysis clearly demonstrated the superiority of PCMM‐Net over existing models, emphasizing its practical applicability in real‐world clinical settings for determining the optimal surgical treatment for individual patients.

## AUTHOR CONTRIBUTIONS

**Hong Zheng:** Investigation (equal); methodology (equal); writing – original draft (lead). **Lian Jian:** Data curation (equal); writing – review and editing (equal). **Li Li:** Formal analysis (equal); validation (equal). **Wen Liu:** Data curation (equal); validation (equal). **Wei Chen:** Conceptualization (equal); methodology (equal); supervision (equal); writing – review and editing (equal).

## CONFLICT OF INTEREST STATEMENT

The authors declare that they have no competing interests.

## ETHICAL STATEMENT

The study protocol was approved by the institutional review board and due to the retrospective nature of the study, the informed consent requirement was abandoned.

## CONSENT

The requirement for informed consent was waived because of the retrospective nature of the study.

## Supporting information


Table S1.

## Data Availability

Information on where data supporting the results reported in the article can be found at Hunan Cancer Hospital, The Affiliated Cancer Hospital of Xiangya School of Medicine, Central South University.

## References

[1] Fotedar V , Fotedar S , Thakur P , Vats S , Negi A , Chanderkant L . Knowledge of breast cancer risk factors and methods for its early detection among the primary health‐care workers in Shimla, Himachal Pradesh. J Educ Health Promot. 2019;8:265. 32002437 10.4103/jehp.jehp_234_19 PMC6967231

[2] Thomssen C , Lüftner D , Untch M , et al. International consensus conference for advanced breast cancer, Lisbon 2019: ABC5 consensus–assessment by a German Group of Experts. Breast Care (Basel). 2020;15(1):82‐95. 32231503 10.1159/000505957 PMC7098316

[3] Li Z , Kang Y . Emerging therapeutic targets in metastatic progression: a focus on breast cancer. Pharmacol Ther. 2016;161:79‐96. 27000769 10.1016/j.pharmthera.2016.03.003 PMC4851893

[4] Çavdar E , İriağaç Y . Predictors of lymphovascular invasion in estrogen receptor positive/Her‐2 negative breast cancer patients treated with neoadjuvant chemotherapy. Turk J Med Sci. 2022;52(4):1111‐1117. 36326379 10.55730/1300-0144.5414 PMC10387951

[5] Zhong YM , Tong F , Shen J . Lympho‐vascular invasion impacts the prognosis in breast‐conserving surgery: a systematic review and meta analysis. BMC Cancer. 2022;22(1):102. 35073848 10.1186/s12885-022-09193-0 PMC8787911

[6] Nijiati M , Aihaiti D , Huojia A , et al. MRI‐based radiomics for preoperative prediction of Lymphovascular invasion in patients with invasive breast cancer. Front Oncol. 2022;12:876624. 35734595 10.3389/fonc.2022.876624 PMC9207467

[7] Zhang J , Wang G , Ren J , et al. Multiparametric MRI‐based radiomics nomogram for preoperative prediction of lymphovascular invasion and clinical outcomes in patients with breast invasive ductal carcinoma. Eur Radiol. 2022;32(6):4079‐4089. 35050415 10.1007/s00330-021-08504-6

[8] Kayadibi Y , Kocak B , Ucar N , Akan YN , Yildirim E , Bektas S . MRI radiomics of breast cancer: machine learning‐based prediction of Lymphovascular invasion status. Acad Radiol. 2022;29:S126‐S134. 10.1016/j.acra.2021.10.026 34876340

[9] Jiang W , Meng R , Cheng Y , et al. Intra‐ and peritumoral based radiomics for assessment of Lymphovascular invasion in invasive breast cancer. J Magn Reson Imaging. 2023:1‐13. 10.1002/jmri.28776 37199241

[10] Wichtmann BD , Harder FN , Weiss K , et al. Influence of image processing on radiomic features from magnetic resonance imaging. Invest Radiol. 2023;58(3):199‐208. 36070524 10.1097/RLI.0000000000000921

[11] Granzier RWY , Ibrahim A , Primakov S , et al. Test‐retest data for the assessment of breast MRI radiomic feature repeatability. J Magn Reson Imaging. 2022;56(2):592‐604. 34936160 10.1002/jmri.28027 PMC9544420

[12] McKinney SM , Sieniek M , Godbole V , et al. International evaluation of an AI system for breast cancer screening. Nature. 2020;577(7788):89‐94. 31894144 10.1038/s41586-019-1799-6

[13] Din NMU , Dar RA , Rasool M , Assad A . Breast cancer detection using deep learning: datasets, methods, and challenges ahead. Comput Biol Med. 2022;149:106073. 36103745 10.1016/j.compbiomed.2022.106073

[14] Kang BJ , Lipson JA , Planey KR , et al. Rim sign in breast lesions on diffusion‐weighted magnetic resonance imaging: diagnostic accuracy and clinical usefulness. J Magn Reson Imaging. 2015;41(3):616‐623. 24585455 10.1002/jmri.24617 PMC7674005

[15] Uematsu T . Focal breast edema associated with malignancy on T2‐weighted images of breast MRI: peritumoral edema, prepectoral edema, and subcutaneous edema. Breast Cancer. 2015;22(1):66‐70. 25336185 10.1007/s12282-014-0572-9

[16] Cheon H , Kim HJ , Lee SM , et al. Preoperative MRI features associated with lymphovascular invasion in node‐negative invasive breast cancer: a propensity‐matched analysis. J Magn Reson Imaging. 2017;46(4):1037‐1044. 28370761 10.1002/jmri.25710

[17] Igarashi T , Furube H , Ashida H , Ojiri H . Breast MRI for prediction of lymphovascular invasion in breast cancer patients with clinically negative axillary lymph nodes. Eur J Radiol. 2018;107:111‐118. 30292254 10.1016/j.ejrad.2018.08.024

[18] Choi BB . Dynamic contrast enhanced‐MRI and diffusion‐weighted image as predictors of lymphovascular invasion in node‐negative invasive breast cancer. World J Surg Oncol. 2021;19:76. 33722246 10.1186/s12957-021-02189-3 PMC7962354

[19] Zhao X , Wan X , Xia W , et al. A cross‐modal 3D deep learning for accurate lymph node metastasis prediction in clinical stage T1 lung adenocarcinoma. Lung Cancer. 2020;145:10‐17. 32387813 10.1016/j.lungcan.2020.04.014

[20] Zheng X , Yao Z , Huang Y , et al. Deep learning radiomics can predict axillary lymph node status in early‐stage breast cancer. Nat Commun. 2020;11:1236. 32144248 10.1038/s41467-020-15027-z PMC7060275

[21] Hua W , Xiao T , Jiang X , et al. Lymph‐vascular space invasion prediction in cervical cancer: exploring radiomics and deep learning multilevel features of tumor and peritumor tissue on multiparametric MRI. Biomed Signal Process Control. 2020;58:101869.

[22] Magny SJ , Shikhman R , Keppke AL . Breast Imaging Reporting and Datasystem. StatPearls Publishing; 2022. 29083600

[23] He K , Zhang X , Ren S , Sun J . Deep residual learning for image recognition. Proc of CVPR. 2016;770‐778.

[24] He K , Zhang X , Ren S , Sun J . Identity mappings in deep residual networks. Proc of ECCV. 2016;630‐645.

[25] Zhang Y , Li K , Li K , Wang L , Zhong B , Fu Y . Image super‐resolution using very deep Residual Channel attention networks. Proc of ECCV. 2018;294‐310.

[26] Hu J , Shen L , Sun G . Squeeze‐and‐excitation networks. Proc of CVPR. 2018;7132‐7141.

[27] Ioffe S , Szegedy C . Batch normalization: accelerating deep network training by reducing internal covariate shift. Proc of ICML. 2015;37:448‐456.

[28] Kingma D , Ba J . Adam: a method for stochastic optimization. Proc of ICLR. 2015:1‐11.

[29] Cheon H , Kim HJ , Kim TH , et al. Invasive breast cancer: prognostic value of peritumoral edema identified at preoperative MR imaging. Radiology. 2018;287(1):68‐75. 29315062 10.1148/radiol.2017171157

