# Supplementary material for: Prior Clinico‐Radiological Features Informed Multi‐Modal MR Images Convolution Neural Network: A novel deep learning framework for prediction of lymphovascular invasion in breast cancer
Source: Cancer Med. 2024 Jan 17;13(3):e6932. doi: 10.1002/cam4.6932 (PMC10905682; doi:10.1002/cam4.6932)
Supplement: Supplementary file 1 — Table S1. [file CAM4-13-e6932-s001.docx]

| Table S1. Comparison of Clinico-radiological Features between the Training and Validation Datasets. | | | | | |
| --- | --- | --- | --- | --- | --- |
| Variables | Total (n = 341) | Training dataset (n = 239) | | Validation dataset (n = 102) | p-value |
| LVI status, n (%) |  |  |  | | 0.984 |
| LVI-negative | 256 (75.1) | 180 (75.3) | 76 (74.5) | |  |
| LVI-positive | 85 (24.9) | 59 (24.7) | 26 (25.5) | |  |
| Age, Median (Q1, Q3) | 51 (45, 58) | 52 (45, 58.5) | 51 (45.2, 57) | | 0.683 |
| Menopausal status, n (%) | |  |  | | 0.751 |
| Premenopausal | 161 (47.2) | 111 (46.4) | 50 (49) | |  |
| Postmenopausal | 180 (52.8) | 128 (53.6) | 52 (51) | |  |
| Location, n (%) |  |  |  | | 0.612 |
| Left | 176 (51.6) | 126 (52.7) | 50 (49) | |  |
| Right | 165 (48.4) | 113 (47.3) | 52 (51) | |  |
| TIC curves, n (%) |  |  |  | | 0.979 |
| Type 1 | 16 (4.7) | 11 (4.6) | 5 (4.9) | |  |
| Type 2 | 133 (39) | 94 (39.3) | 39 (38.2) | |  |
| Type 3 | 192 (56.3) | 134 (56.1) | 58 (56.9) | |  |
| FGT density, n (%) | |  |  | | 0.789 |
| Dense | 73 (21.4) | 50 (20.9) | 23 (22.5) | |  |
| Heterogeneously dense | 118 (34.6) | 86 (36) | 32 (31.4) | |  |
| Scattered | 104 (30.5) | 73 (30.5) | 31 (30.4) | |  |
| Predominantly fatty | 46 (13.5) | 30 (12.6) | 16 (15.7) | |  |
| BPE, n (%) |  |  |  | | 0.907 |
| None/minimal | 91 (26.7) | 64 (26.8) | 27 (26.5) | |  |
| Mild | 147 (43.1) | 104 (43.5) | 43 (42.2) | |  |
| Moderate | 66 (19.4) | 47 (19.7) | 19 (18.6) | |  |
| Marked | 37 (10.9) | 24 (10) | 13 (12.7) | |  |
| Intratumoral high signal intensity, n (%) | |  |  | | 1.000 |
| Absence | 236 (69.2) | 165 (69) | 71 (69.6) | |  |
| Presence | 105 (30.8) | 74 (31) | 31 (30.4) | |  |
| Peritumoral edema, n (%) | |  |  | | 0.897 |
| Absence | 234 (68.6) | 163 (68.2) | 71 (69.6) | |  |
| Presence | 107 (31.4) | 76 (31.8) | 31 (30.4) | |  |
| Subcutaneous edema, n (%) | |  |  | | 0.614 |
| Absence | 278 (81.5) | 197 (82.4) | 81 (79.4) | |  |
| Presence | 63 (18.5) | 42 (17.6) | 21 (20.6) | |  |
| Intratumoral necrosis, n (%) | |  |  | | 0.82 |
| Absence | 275 (80.6) | 194 (81.2) | 81 (79.4) | |  |
| Presence | 66 (19.4) | 45 (18.8) | 21 (20.6) | |  |
| Internal enhancement pattern, n (%) | |  |  | | 0.896 |
| Homogeneous | 272 (79.8) | 192 (80.3) | 80 (78.4) | |  |
| Heterogeneous | 66 (19.4) | 45 (18.8) | 21 (20.6) | |  |
| Rim enhancement | 3 (0.9) | 2 (0.8) | 1 (1) | |  |
| Adjacent vessel sign, n (%) | |  |  | | 1.000 |
| Absence | 135 (39.6) | 95 (39.7) | 40 (39.2) | |  |
| Presence | 206 (60.4) | 144 (60.3) | 62 (60.8) | |  |
| Increased ipsilateral vascularity, n (%) | |  |  | | 0.186 |
| Absence | 187 (54.8) | 125 (52.3) | 62 (60.8) | |  |
| Presence | 154 (45.2) | 114 (47.7) | 40 (39.2) | |  |
| mrALN status, n (%) | |  |  | | 1.000 |
| Absence | 270 (79.2) | 189 (79.1) | 81 (79.4) | |  |
| Presence | 71 (20.8) | 50 (20.9) | 21 (20.6) | |  |
| Short-axis diameter of largest ALN, Median (Q1, Q3) | 0.5 (0.3, 0.8) | 0.5 (0.3, 0.8) | 0.5 (0.3, 0.8) | | 0.6 |
| DWI rim sign, n (%) | |  |  | | 0.732 |
| Absence | 250 (73.3) | 177 (74.1) | 73 (71.6) | |  |
| Presence | 91 (26.7) | 62 (25.9) | 29 (28.4) | |  |
| ***Abbreviation:*** TIC, time-signal intensity; FGT, fibroglandular tissue; BPE, breast parenchymal enhancement; mrALN, MRI-reported axillary lymph node; DWI, diffusion weighted imaging; LVI, lymphovascular invasion | | | | | |
